# Long-term Follow-up Optical Coherence Tomography Assessment of Primary Percutaneous Coronary Intervention for Unprotected Left Main

**DOI:** 10.31083/j.rcm2512445

**Published:** 2024-12-19

**Authors:** Zlatko Mehmedbegovic, Vladan Vukcevic, Sinisa Stojkovic, Branko Beleslin, Dejan Orlic, Miloje Tomasevic, Miodrag Dikic, Milorad Tesic, Dejan Milasinovic, Srdjan Aleksandric, Vladimir Dedovic, Milorad Zivkovic, Stefan Juricic, Dario Jelic, Djordje Mladenovic, Goran Stankovic

**Affiliations:** ^1^Department of Cardiology, University Clinical Center of Serbia, 11000 Belgrade, Serbia; ^2^Faculty of Medicine, University of Belgrade, 11000 Belgrade, Serbia; ^3^Faculty of Medical Sciences, University of Kragujevac, 34000 Kragujevac, Serbia

**Keywords:** OCT, unprotected left main, primary PCI, long-term follow-up, strut endothelization, stent malapposition

## Abstract

**Background:**

Elective unprotected left main (ULM) percutaneous coronary intervention (PCI) has long-term mortality rates comparable to surgical revascularization, thanks to advances in drug-eluting stent (DES) design, improved PCI techniques, and frequent use of intravascular imaging. However, urgent PCI of ULM culprit lesions remains associated with high in-hospital mortality and unfavourable long-term outcomes, including DES restenosis and stent thrombosis (ST). This analysis aimed to examine the long-term outcomes and healing of DES implanted in ULM during primary PCI using high-resolution optical coherence tomography (OCT) imaging.

**Methods:**

A total of 15 consecutive patients undergoing long-term OCT follow-up of ULM primary PCI from a high-volume center were included in this analysis. During the index primary PCI all subjects underwent angio-guided DES implantation, and follow-up was uneventful in all but one subject who had a non-target PCI lesion. The primary endpoint was the percentage of covered, uncovered, and malappossed stent struts at long-term follow-up. Secondary endpoints included quantitative and qualitative OCT measurements. For the left main bifurcation, a separate analysis was performed for three different segments: left main (LM), polygon of confluence (POC) and distal main branch (dMB), in all cases.

**Results:**

The average follow-up interval until OCT was 1580 ± 1260 days. Despite aorto-ostial stent protrusions in 40% of patients, optimal image quality was achieved in 93.3% of cases. There were higher rates of malapposed (11.4 ± 16.6 vs. 13.1 ± 8.3 vs. 0.3 ± 0.5%; *p* < 0.001) and lower rates of covered struts (81.7 ± 16.8 vs. 83.7 ± 9.2 vs. 92.4 ± 6.8%; *p* = 0.041) observed for the LM and POC segment compared to the dMB. Significantly malapposed stent struts (>400 μm) were less likely to be covered at follow-up, than struts with a measured strut to vessel wall distance of <400 μm (15.4 ± 21.6 vs. 24.8 ± 23.9%; *p* = 0.011). Neoatherosclerosis was observed in 5 (33.3%) and restenotic neointimal hyperplasia (NIH) in 2 (13.3%) patients, requiring PCI in 33.3% of patients.

**Conclusions:**

Long-term OCT examination of DES implanted during primary PCI for culprit ULM lesions demonstrated high rates of incomplete strut coverage, late malapposition, and high subclinical DES failure rates. These negative OCT results highlight the need for image optimization strategies during primary PCI to improve DES-related long-term outcomes.

## 1. Introduction

Elective percutaneous coronary interventions (PCI) on the unprotected left main (ULM) are increasing in popularity thanks to improved stent design, optimization of PCI techniques and increasing use of intravascular imaging (IVI), and have long-term mortality comparable to surgical revascularization [1]. On the other hand, Urgent PCI of the culprit lesion in the ULM segment still has high in-hospital mortality rates despite immediate reperfusion, mainly due to a large amount of myocardium at risk [2, 3]. Furthermore, the long-term outcomes of these patients after hospital discharge are still not comparable to those of patients undergoing elective ULM PCI, with higher rates of restenosis and thrombosis [4, 5]. To ensure favourable long-term outcomes after drug-eluting stent (DES) implantation, procedural IVI guidance is recommended to optimize stent implantation, particularly stent expansion and good vessel wall opposition, thereby promoting endothelialization and long-term device patency [6]. Angiographically undetectable stent malapposition occurs more frequently in large coronary anatomies, e.g., the left main bifurcation segment, and after PCI with complex two-stent techniques [7, 8, 9, 10, 11]. Despite its recognized benefit, reported rates of imaging during primary PCI-ULM procedures are low, particularly due to the clinical circumstances with impending circulatory collapse that mandate prompt stent implantation and rapid restoration of flow [3]. Consequently, reports on short- and long-term IVI evaluations of angio-guided ULM stenting during primary PCI are lacking. Therefore, we performed long-term optical coherence tomography (OCT) to investigate long-term DES healing in patients with uneventful clinical follow-up, who underwent angio-guided ULM primary PCI.

## 2. Method

### 2.1 Study Population

**Fig. 1.**
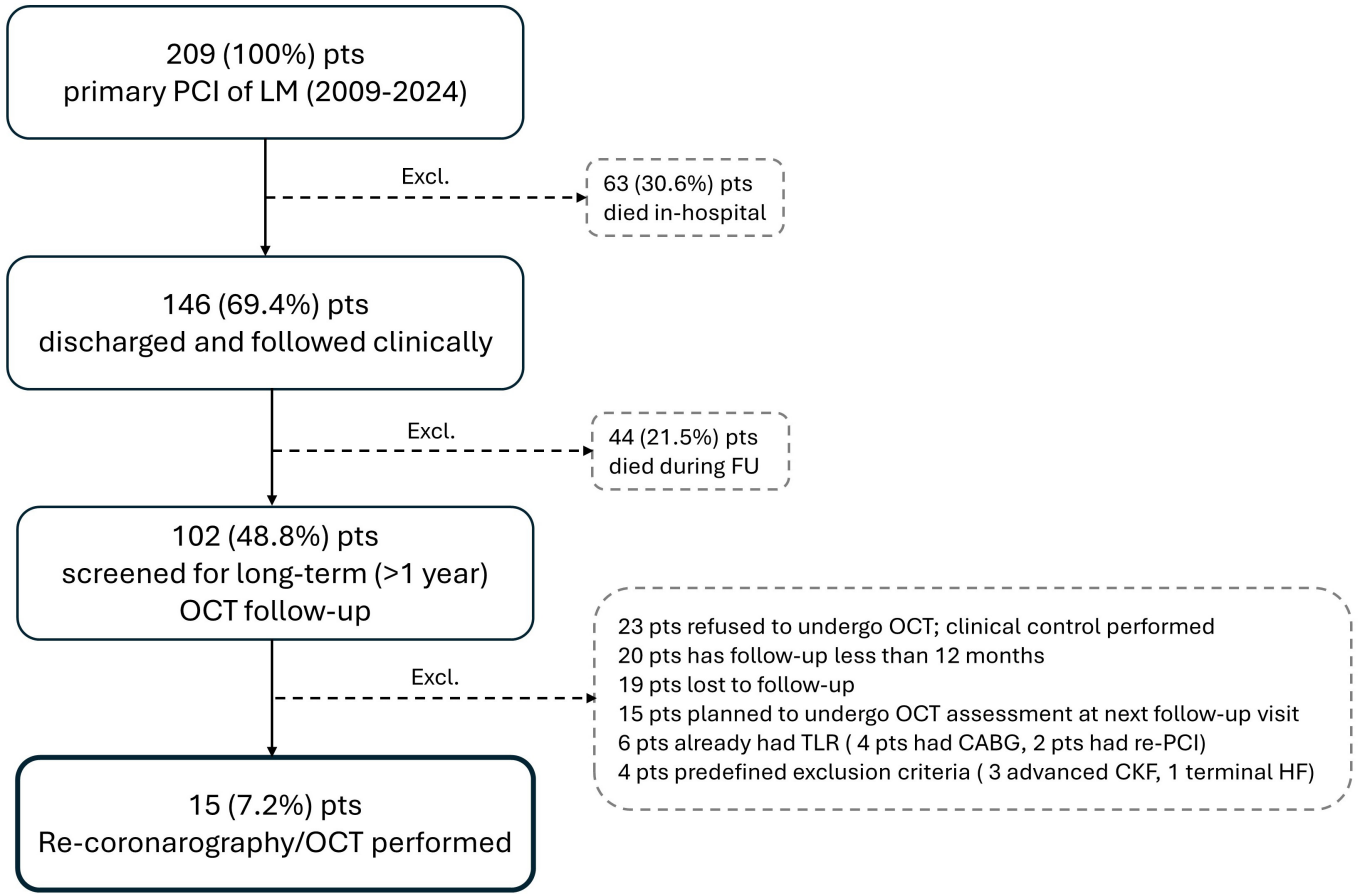
**Patient flow chart**. CABG, coronary artery bypass graft; CKF, chronic kidney failure; Excl., excluded patients; FU, follow-up; LM, left main; HF, heart failure; OCT, optical coherence tomography; PCI, percutanous coronary intervention; pts, patients; TLR, target lesion revascularization.

A total of 15 consecutive patients who underwent primary PCI for a culprit ULM lesion (from November 2012 to August 2023) in a high-volume tertiary center (University Clinical Center of Serbia, Belgrade, Serbia) and who had long-term follow-up OCT imaging were included in this analysis (Fig. 1). All patients underwent angio-guided DES implantation during primary PCI. Exclusion criteria for OCT follow-up included heart failure with left ventricular ejection fraction <30%, chronic kidney disease (serum creatinine >2 mg/dL), renal dialysis, known allergy to antiplatelet agents or contrast media. The study protocol was approved by the institutional research board and informed consent was obtained for each patient before any procedure was performed.

### 2.2 OCT Image Acquisition, Analysis, and Definitions

A conventional angioplasty guidewire (0.014 inch) was advanced distally to the region of interest, then the OCT catheter (Dragonfly Optis, Abbott, Santa Clara, CA, USA) was advanced over the guidewire at least 10 mm beyond the region of interest. The images were calibrated by automatic adjustment of the Z-offset and the automatic pullback was set to 20 mm, or 10 mm/s. Data were acquired and digitally stored using a commercially available OCT system (C7-XR, OCT Imaging System, Abbott, Santa Clara, CA, USA) after intracoronary administration of 50 to 200 mm of nitroglycerin via conventional guiding catheters. During image acquisition, blood was displaced by injecting a hypo- or iso-osmolar contrast agent using a power injector or hand injection. OCT pullbacks were performed from the distal main branch to the ostial part of the left main (LM). OCT analyses were performed using the dedicated software CASS Intravascular, service pack 2.1 (PIE Medical, Maastricht, The Netherlands) with semi-automatic contour and strut detection functions. Quantitative and qualitative OCT analysis of the lumen, stent, and strut was performed along the entire stented segment for each recorded cross section at 0.2 mm slices. All cross-sectional images were initially quality screened and assessed, and sections with any portion of the stent beyond the image field of view, or if the image had poor quality caused by residual blood, artefacts, or reverberations, were excluded from the analysis. Any strut with an ambiguous appearance was also not included in the qualitative analysis [10].

**Fig. 2.**
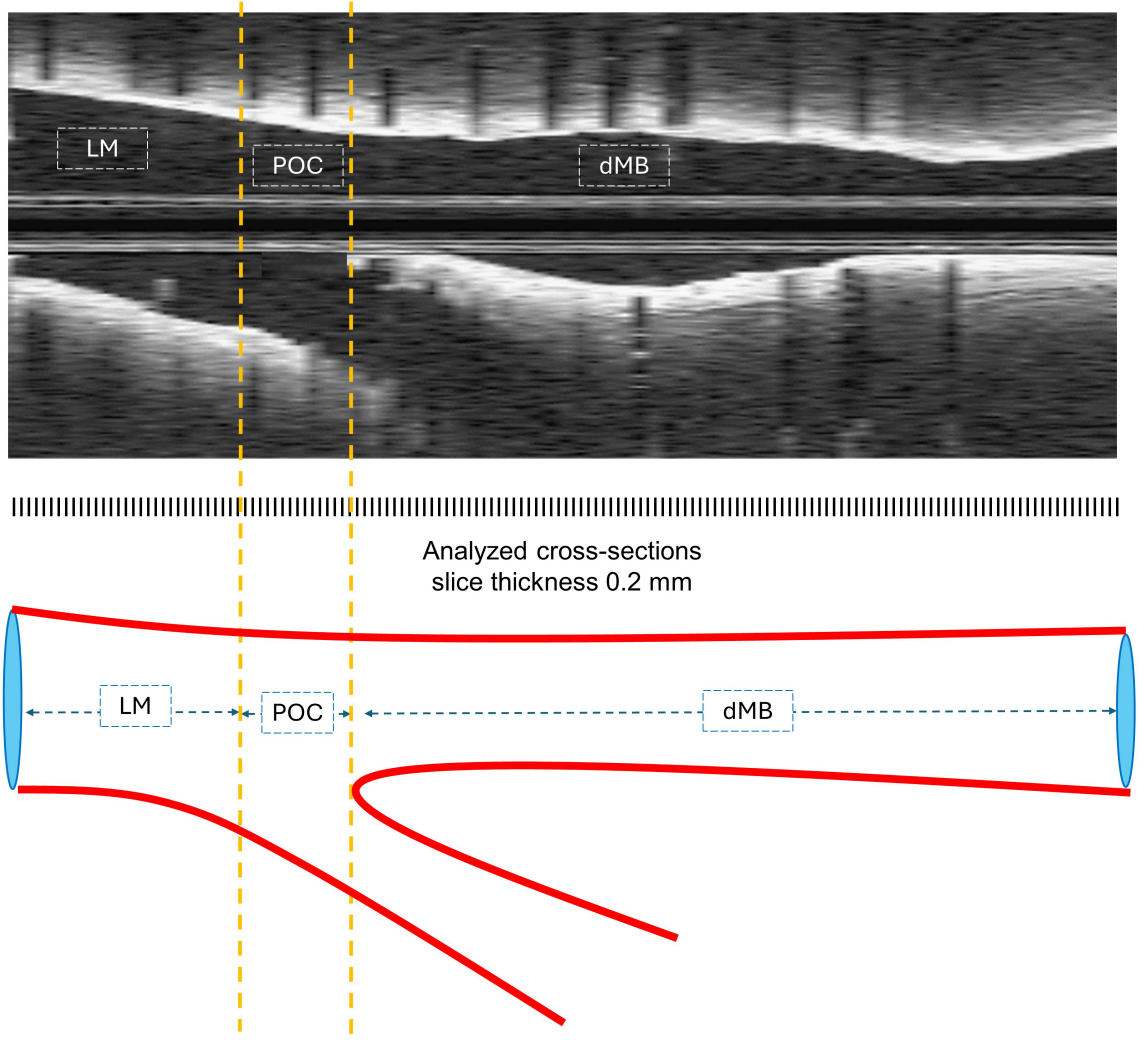
**OCT longitudinal pullback and corresponding illustration of 3 segments of anatomical differentiation**. dMB, distal main branch; LM, left main; OCT, optical coherence tomography; POC, polygon of confluence.

The analysis was stratified according to the underlying bifurcation anatomy and arbitrary identification of three subsegments as recommended by consensus papers: LM segment (LM), polygon of confluence (POC), and distal main branch (dMB) (Fig. 2) [12].

For two patients who underwent double stenting, side branch pullbacks were also performed, but were not included in this analysis. All cross-sectional images were initially reviewed for quality assessment and excluded from analysis if any part of the stent was off-screen or if the image was of poor quality due to residual blood, artifacts, or reverberation. Struts were defined as covered, only if they were completely covered by neointimal tissue, while partially covered struts were considered uncovered according to previous histological reports [13]. Malapposed struts were defined as all struts that were not in contact with the lumen with measured malapposition distance greater than strut thickness + polymer thickness [10]. Struts were considered to be significantly malapposed if measured malapposition distance was >400 µm [14, 15, 16]. Malapposed struts were further stratified as covered or uncovered, according to neointimal coverage, as specified above.

**Fig. 3.**
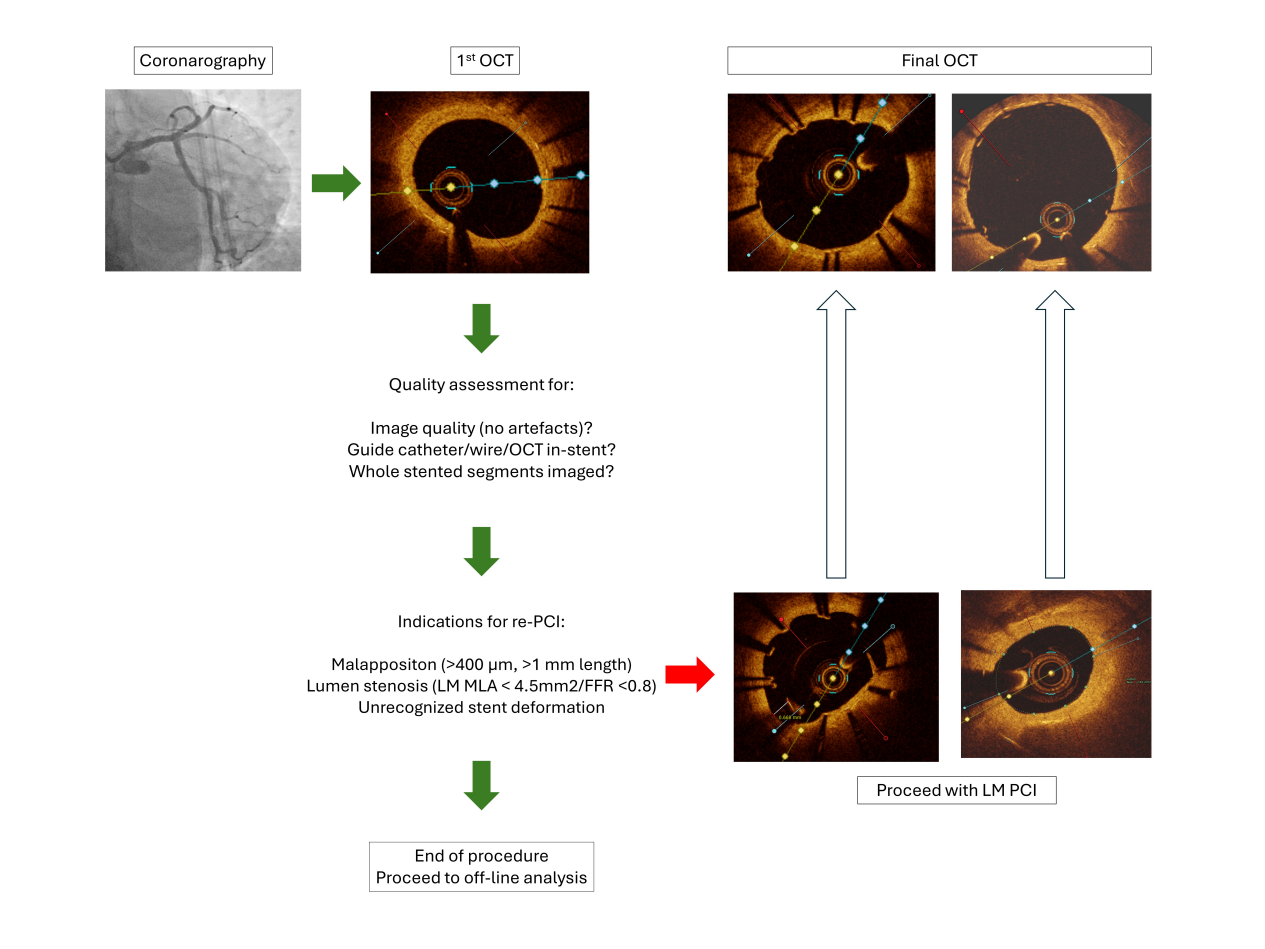
**OCT follow-up protocol**. LM, left main; MLA, minimal lumen area; OCT, optical coherence tomography; PCI, percutaneous coronary intervention; FFR, fractional flow reserve.

OCT recording and *on-line* analysis was performed according to a prespecified protocol, as shown in Fig. 3.

### 2.3 Endpoints

The primary OCT endpoints were the percentage of covered, uncovered, and malapposed stent struts assessed by OCT at long-term follow-up by segment (LM/POC/dMB). The main secondary endpoint was the impact of malapposition distance on vascular response and strut coverage. Additional secondary endpoints included feasibility of OCT imaging, lumen, stent measurements, cross-sectional and volumetric measurements, extent of neointimal hyperplasia (NIH), incidence of neoatherosclerosis, and impact of stent optimization techniques, performance of proximal optimization technique (POT) and kissing balloons inflation (KBI). For a complete list of OCT endpoints and definitions, see the OCT **Supplementary Material** (OCT variable and endpoints definitions).

### 2.4 Statistical Analysis

Statistical analysis was performed using SPSS software version 26.0 (IBM Corp., Armonk, NY, USA). Continuous data were expressed as mean ± standard deviation. Comparisons of categorical data were made using χ-square statistics or Fisher’s exact test. Student’s *t*-test, paired *t*-test, or Wilcoxon signed-rank test were used to compare continuous variables. Comparisons among the three bifurcation segments were performed using one-way analysis of variance (ANOVA). A *p*-value < 0.05 was considered statistically significant.

## 3. Results

### 3.1 Baseline Primary PCI

Baseline clinical, angiographic, primary PCI procedural, and hospitalization characteristics of 15 patients that underwent urgent angiography with primary PCI of the ULM are reported in Table 1.

**Table 1.** **Baseline primary PCI characteristics**.

Baseline and angiographic characteristics		N = 15	Procedural and post-PCI characteristics	N = 15
Age (mean ± SD)		59.7 ± 9.1	Mechanical circulatory support (N, %)	0 (0.0)
Male (N, %)		12 (80.0)	“MADS” stenting classification (N, %)	
STEMI (N, %)		9 (60.0)	A	14 (93.3)
Admission KILLIP class (N, %)			S	1 (6.7)
	1	14 (93.3)	Single stent	13 (86.7)
	4	1 (6.7)		
Total ischemic time (mean ± SD)		365.6 ± 258.3	Two stents	2 (13.3)
FMC to PCI time (mean ± SD)		186.1 ± 174.9	TAP	1 (6.7)
Procedure time (mean ± SD)		54.8 ± 32.1	Double-kissing crush	1 (6.7)
Femoral access (N, %)		5 (33.3)	Immediate PCI of other lesions (N, %)	2
Catheter >6 Fr (N, %)		3 (20.0)	No of stents implanted (mean ± SD)	1.5 ± 0.8
Coronary left dominance (N, %)		3 (20.0)	No of wires used (mean ± SD)	2.3 ± 0.8
Collaterals > Rentrop 1 (N, %)		1 (6.7)	No of balloons used (mean ± SD)	3.8 ± 2.7
Diseased vessels (N, %)			TIMI 3 final post PCI (N, %)	
	Isolated LM disease	8 (53.3)	LM	15 (100.0)
	LM + 1 vessel	4 (26.7)	distal MB	15 (100.0)
	LM + 2 vessels	1 (6.7)	SB	15 (100.0)
	LM + 3 vessels	2 (13.3)	Bifurcation lesion success (N, %)	
“MEDINA” classification (N, %)			Overall	14 (93.3)
	1.1.0	4 (26.7)	MB success	15 (100.0)
	1.1.1	4 (26.7)	SB success	14 (93.3)
	1.0.0	3 (20.0)	Post PCI EF (mean ± SD)	45.1 ± 14.4
	0.1.0	2 (13.3)	Mechanical ventilatory support (N, %)	1 (6.7)
	0.1.1	1 (6.7)	KILLIP 4 class during hospitalization (N, %)	3 (20.0)
	1.1.0.1	1 (6.7)	Days of ICU stay (mean ± SD)	8.3 ± 10.6
TIMI flow 0–1 pre-PCI (N, %)			Discharge P2Y12 inhibitor (N, %)	
	LM	3 (20.0)	Clopidogrel	3 (20.0)
	distal MB	5 (33.3)	Ticagrelor	11 (73.3)
	SB	4 (26.7)	Prasugrel	1 (6.7)

PCI, percutaneous coronary intervention; STEMI, ST-elevation myocardial infarction; FMC, first medical contact; LM, left main; EF, ejection fraction; ICU, intensive care unit; MADS, M-main, A-across, D-distal, S-side; MB, main branch; TIMI, thrombolysis in myocardial infarction; SB, side branch; SD, standard deviation; TAP, T-And Protrusion.

Most patients had a diagnosis of ST-elevation myocardial infarction (STEMI) as the indication for the PCI, had no signs of acute heart failure before the primary PCI (93.3%), underwent a simple, provisional stenting strategy in 86.7% of patients. In two cases, complex bifurcation stenting T-And Protrusion (TAP), and Double-Kissing Crush (DKC) were performed. The bifurcation procedure was successful in 14 patients (93.3%). All procedures were angiographically guided and without mechanical circulatory support. All subjects had an uncomplicated post-procedural intensive unit stay and were discharged with potent P2Y12 inhibitors in 80% of cases. After the initial discharge, only one subject had a myocardial infarction and underwent a PCI with non-target lesion revascularization (non-TLR), while the other subjects had an uneventful follow-up.

### 3.2 Follow-up OCT

Follow-up angiography and OCT were performed 1580.5 ± 1206.4 days after the primary PCI (interquartile range (IQR) 478-2138) (Table 2).

**Table 2.** **OCT feasibility analysis and qualitative endpoints**.

		N = 15
Days from baseline PCI (N, mean ± SD)		1580.5 ± 1206.4
OCT image quality optimal for analysis (N, %)		14 (93.3)
Amount of contrast dye used for follow-up procedure (mL, ± SD)		240.7 ± 74.3
Difficult guide catheter selective LM canulation (N, %)		3 (20.0)
OCT/wire inadvertent abluminal stent side crossing (N, %)		4 (26.6)
Left main ostial segment visible (N, %)		7 (46.7)
Stent aortal protrusion (N, %)		6 (40.0)
	Major	3 (20.0)
	Minor	3 (20.0)
Unanalysable ostial stent segment (N, %)		5 (33.3)
Unanalysable stent measurable length (mm, mean ± SD)		1.7 ± 0.2
Longitudinal stent deformation (N, %)		1 (6.7)
SB overhanging struts (N, %)		14 (93.3)
Neointimal proliferation on overhanging struts (N, %)		10 (66.7)
Obstruction of SB ostium, maximal neointimal proliferation distance ratio to SB opening (%, mean ± SD)		63.9 ± 21.5
Thrombus (N, %)		2 (13.4)
White (N, %)		0 (0.0)
Red (N, %)		0 (0.0)
Organized (N, %)		2 (13.4)
Neoatherosclerosis (N, %)		5 (33.3)
Calcific neoatherosclerosis (N, %)		2 (13.4)
PCI following OCT/coronarography (N, %)		7 (46.7)
TLR only (N, %)		2 (13.4)
Non-TLR (N, %)		2 (13.4)
TLR and Non-TLR (N, %)		3 (20.0)

OCT, optical coherence tomography; PCI, percutaneous coronary intervention; LM, left main; SB, side branch; SD, standard deviation; TLR, target lesion revascularization.

**Fig. 4.**
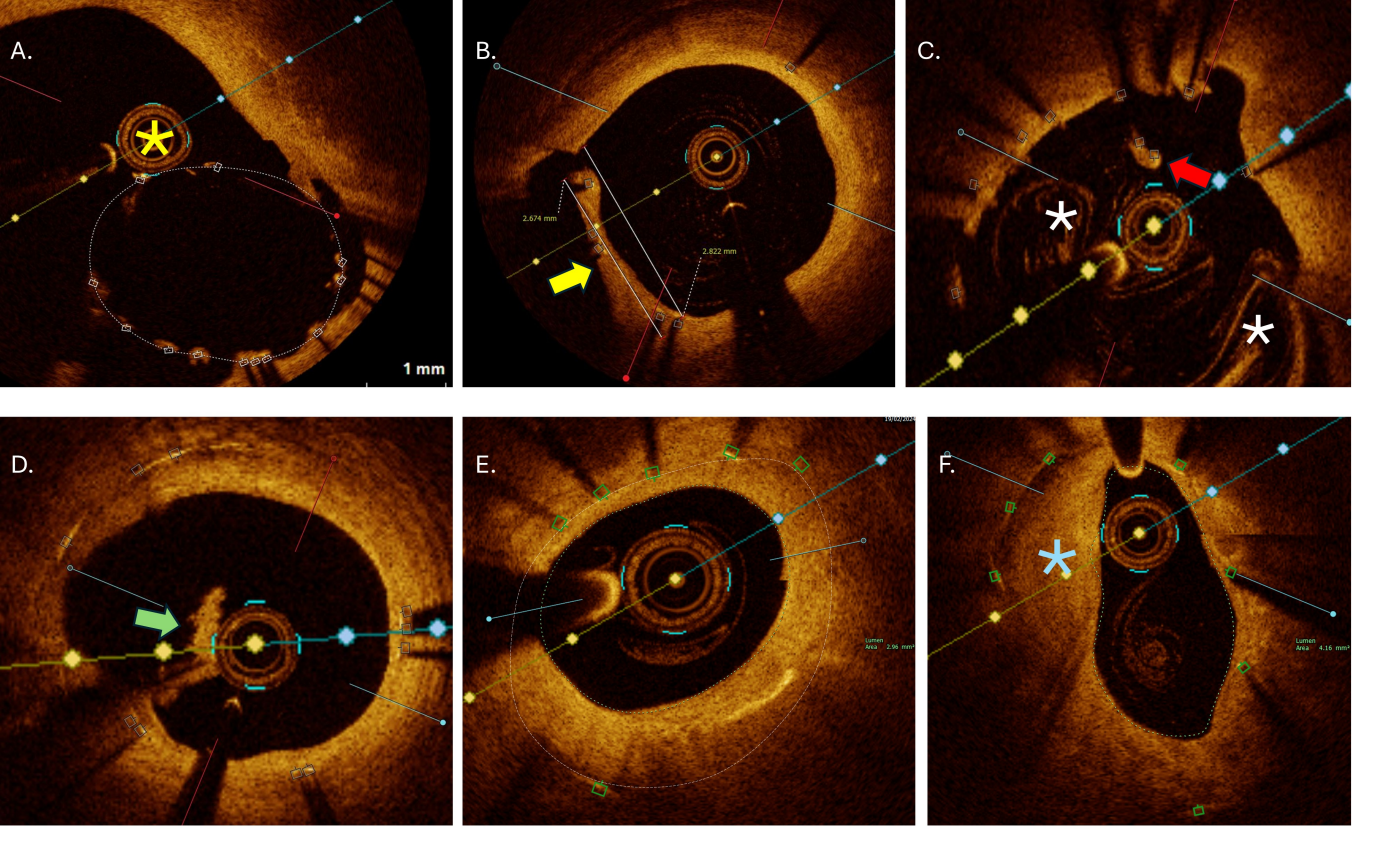
**OCT exemplary images of analysed qualitative endpoints**. (A) Passage of OCT catheter (yellow asterix) outside the stent in large polygon of confluence region due to suboptimal baseline balloon proximal optimization. (B) Obstruction of side branch orifice by overhanging struts neointimal hyperplasia (yellow arrow) with measurements. (C) Longitudinal stent deformation at the level of proximal stent edge with covered floating struts (red arrow). Residual blood artefacts (white asterix) characteristic for ostial cross-sections. (D) Organized thrombi (green arrow) attached on uncovered struts belonging to metallic neocarina following T and protrusion bifurcation stenting. (E) Significant neointimal hyperplasia with minimal lumen area of 2.96 mm^2^ within under-expanded stent (area 5.2 mm^2^) located at mid shaft of left main. (F) In-stent neoatherosclerosis (blue asterisk) with peri-strut low intensity area, located in distal left main with minimal lumen area 4.16 mm^2^. OCT, optical coherence tomography.

Despite stent protrusion following initial ostial stenting (40%), and abluminal wire and/or OCT catheter placement (26.6%) (Fig. 4A), optimal image quality runs were obtained in 93.3% (one subject had moderate quality due to residual blood swirling artefacts), with an unanalysable measured stent length of 1.7 ± 0.2 mm, located in all cases at the proximal stent edge. Overhanging (jailing) struts were detected in almost all cases (93.3%), with neointimal proliferation in 10 cases (66.7%) (Fig. 4B), while 2 subjects had evidence of organized thrombi attached to partially covered struts (Fig. 4D). Evidence of neoatherosclerosis was observed in 5 subjects (Fig. 4E,F), with 7 (46.7%) patients undergoing ad-hoc PCI, of which 5 were target lesion revascularization (TLR) (33.3%).

### 3.3 OCT Quantitative Analysis

A total of 1944 cross-sections (frames) and 16,520 individual struts was analysed. Larger mean lumen (12.8 ± 4.9 vs. 12.7 ± 3.7 vs. 7.6 ± 1.9 mm^2^; *p* < 0.001) and stent area (13.2 ± 3.7 vs. 11.8 ± 3.9 vs. 9.0 ± 1.9 mm^2^; *p* = 0.004) were measured in LM and POC region compared to the dMB segment. While mean NIH area was not significant between segments (1.6 ± 0.8 vs. 1.6 ± 0.8, vs. 1.4 ± 0.5 mm^2^; *p* = 0.779), significant differences in total malapposition volume (3.5 ± 3.8 vs. 5.8 ± 3.9 vs. 0.7 ± 0.7 mm^3^; *p* = 0.001) and mean malapposed area were obtained (1.7 ± 2.9 vs. 1.9 ± 1.5 vs. 0.2 ± 0.6 mm^2^; *p* = 0.037) across segments. The LM and POC had greater absolute difference in reference area-mean stent area (3.9 ± 4.2 and 4.5 ± 2.8 mm^2^) compared to the distal segment (–1.7 ± 1.8 mm^2^; *p* < 0.001). Larger lumen area stenosis (39.1 ± 23.2 and 36.3 ± 25.0%) and smaller stent expansion (63.8 ± 25.7 and 56.9 ± 15.3%) were also observed for proximal segments, LM and POC, compared to the dMB (lumen stenosis 16.4 ± 26.5%; *p* = 0.034, and stent expansion 100.9 ± 18.6%; *p* < 0.001).

### 3.4 Strut Coverage Analysis

Detailed strut-level analysis showed overall strut coverage of 89.5 ± 7.3%, while 4.4 ± 3.0, and 1.2 ± 0.7% of struts were malapposed <400 µm and >400 µm, respectively, for the whole analysed stented segment. The percentage of covered and malapposed struts were significantly different for the LM and POC compared to the dMB bifurcation segments (Table 3).

**Table 3.** **Lumen, stent and strut quantitative OCT analysis**.

	All segments	LM	POC	dMB	*p*
Number of analysed cross sections (N, mean ± SD)	129.6 ± 51.6	31.1 ± 21.4	11.3 ± 6.1	64.0 ± 45.2	<0.001
Total region of interest length (cm, mean ± SD)	25.8 ± 8.4	NA	NA	NA	NA
Analysed stent length (cm, mean ± SD)	21.0 ± 7.4	6.6 ± 4.6	2.4 ± 0.8	12.4 ± 7.0	<0.001
Reference area (mm^2^, mean ± SD)	NA	16.4 ± 4.9	16.4 ± 4.9	7.3 ± 2.0	<0.001
MLA (mm^2^, mean ± SD)	5.3 ± 1.9	10.3 ± 5.2	9.8 ± 3.9	5.9 ± 1.8	<0.001
Minimal lumen diameter at MLA (mm, mean ± SD)	2.2 ± 0.5	2.9 ± 0.9	2.9 ± 0.5	2.4 ± 0.5	0.051
Mean lumen area (mm^2^, mean ± SD)	9.4 ± 2.1	12.8 ± 4.9	12.7 ± 3.7	7.6 ± 1.9	<0.001
Mean minimal lumen diameter (mm, mean ± SD)	2.9 ± 0.3	3.5 ± 0.6	2.8 ± 0.4	2.8 ± 0.4	0.024
Lumen area stenosis (%, mean ± SD)	NA	39.1 ± 23.2	36.3 ± 25.0	16.4 ± 26.5	0.034
Lumen volume (mm^3^, mean ± SD)	198 ± 71.9	75.3 ± 43.4	31.0 ± 14.4	95.1 ± 57.9	0.001
Stent volume (mm^3^, mean ± SD)	210.3 ± 76.7	79.4 ± 48.6	27.3 ± 12.8	10.8 ± 66.9	<0.001
In-stent NIH volume (mm^3^, mean ± SD)	30.0 ± 14.0	10.5 ± 9.9	3.6 ± 1.8	17.6 ± 13.9	0.002
NIH area at MLA (mm^2^, mean ± SD)	2.5 ± 1.8	1.9 ± 1.1	1.9 ± 0.9	1.9 ± 1.3	0.961
Mean NIH area (mm^2^, mean ± SD)	1.5 ± 0.6	1.6 ± 0.8	1.6 ± 0.8	1.4 ± 0.5	0.779
Malapposition volume (mm^3^, mean ± SD)	11.6 ± 9.1	3.5 ± 3.8	5.8 ± 3.9	0.7 ± 0.7	<0.001
Mean malapposition area (mm^2^, mean ± SD)	0.4 ± 0.2	1.7 ± 2.9	1.9 ± 1.5	0.2 ± 0.6	0.037
Mean stent area (mm^2^, mean ± SD)	10.3 ± 1.8	13.2 ± 3.7	11.8 ± 3.9	9.0 ± 1.9	0.004
Stent area at MLA (mm^2^, mean ± SD)	8.0 ± 2.0	11.5 ± 4.1	11.1 ± 3.5	8.1 ± 1.9	0.016
Minimal stent area (mm^2^, mean ± SD)	7.0 ± 1.9	10.3 ± 3.5	8.7 ± 1.8	7.1 ± 1.9	0.006
Minimal stent expansion (%, mean ± SD)	NA	63.8 ± 25.7	56.9 ± 15.3	100.9 ± 18.6	<0.001
Mean stent expansion (%, mean ± SD)	NA	81.6 ± 21.5	74.7 ± 16.1	128.4 ± 34.2	<0.002
Difference reference area - mean stent area (mm^2^, mean ± SD)	NA	3.9 ± 4.2	4.5 ± 2.8	–1.7 ± 1.8	<0.001
Total number of analysed struts (N, mean ± SD)	1101.3 ± 762.3	289.1 ± 255.3	100.1 ± 41.9	756.3 ± 725.2	0.001
Analyzed struts per cross section (N, mean ± SD)	10.1 ± 2.1	9.1 ± 2.8	9.3 ± 1.7	11.2 ± 2.8	0.043
Covered (%, mean ± SD)	86.9 ± 7.0	78.9 ± 19.2	79.8 ± 10.5	92.3 ± 6.8	0.015
Uncovered (%, mean ± SD)	8.7 ± 4.9	9.7 ± 8.6	7.0 ± 4.9	7.3 ± 6.5	0.526
Malapposed (%, mean ± SD)	4.4 ± 3.0	11.4 ± 16.6	13.1 ± 8.3	0.3 ± 0.5	0.005
Malapposed and uncovered (%, mean ± SD)	2.6 ± 2.5	8.6 ± 13.1	9.3 ± 6.3	0.2 ± 0.5	0.01
Malapposition >400 µm (%, mean ± SD)	1.7 ± 1.2	5.6 ± 10.3	8.1 ± 6.7	0.0 ± 0.0	0.011
Malapposition >400 µm and uncovered (%, mean ± SD)	1.2 ± 0.7	4.4 ± 8.0	5.9 ± 4.6	0.0 ± 0.0	0.013
Total covered (covered + malapposed covered) (%, mean ± SD)	89.5 ± 7.3	81.7 ± 16.8	83.7 ± 9.2	92.4 ± 6.8	0.041
Cross sections with >30% uncovered struts (%, mean ± SD)	7.9 ± 6.3	9.9 ± 12.4	3.8 ± 6.6	5.5 ± 7.9	0.204
Maximum consecutive length with uncovered struts (%, mean ± SD)	2.8 ± 2.0	1.3 ± 1.9	0.8 ± 0.5	1.6 ± 1.5	0.311
Cross sections with >30% malapposed struts (%, mean ± SD)	7.3 ± 4.6	20.6 ± 32.2	19.9 ± 17.8	0.0 ± 0.0	0.018
Maximum consecutive length with malapposed struts (%, mean ± SD)	1.9 ± 1.3	1.2 ± 1.6	1.6 ± 1.5	0.2 ± 0.2	0.024

OCT, optical coherence tomography; LM, left main; POC, polygon of confluence; dMB, distal main branch; MLA, minimal lumen area; NA, not applicable; NIH, neointimal hyperplasia; SD, standard deviation.

**Fig. 5.**
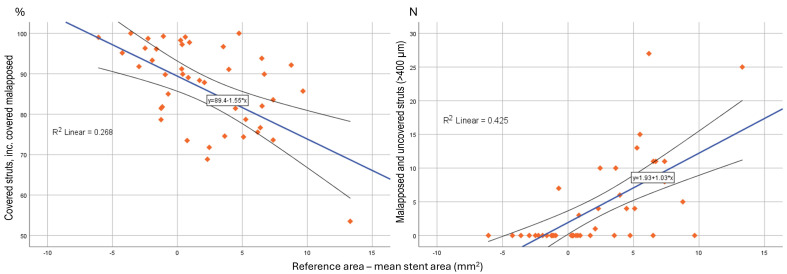
**Scatter-dot matrix showing and correlation analysis between absolute difference, reference - mean stent area, to strut coverage and malapposition**.

Absolute difference in reference area and mean stent area correlated with percentage of covered and malapposed >400 µm and uncovered struts, across segments (Table 4 and Fig. 5).

**Table 4.** **Correlation analysis of absolute difference reference area and mean stent area and strut coverage and malapposition**.

	LM	POC	dMB	R^2^	*p*
Reference area (mm^2^, mean ± SD)	16.4 ± 4.9	16.4 ± 4.9	7.3 ± 2.0	NA	<0.001
Mean stent area (mm^2^, mean ± SD)	13.2 ± 3.7	11.8 ± 3.9	9.0 ± 1.9	NA	0.004
Difference reference area - mean stent area (mm^2^, mean ± SD)	3.9 ± 4.2	4.5 ± 2.8	–1.7 ± 1.8	NA	<0.001
Total covered (%, mean ± SD)	78.9 ± 19.2	79.8 ± 10.5	92.3 ± 6.8	0.268	0.000
Malapposed (N, mean ± SD)	25.2 ± 45.4	13.1 ± 9.5	19.3 ± 33.3	0.355	0.241
Malapposed (%, mean ± SD)	11.4 ± 16.6	13.1 ± 8.3	0.3 ± 0.5	0.265	0.008
Malapposed and uncovered (N, mean ± SD)	20.0 ± 41.2	9.4 ± 8.4	0.0 ± 0.0	0.316	0.429
Malapposed and uncovered (%, mean ± SD)	8.6 ± 13.1	9.3 ± 6.3	0.2 ± 0.5	0.252	0.161
Malapposed >400 µm (N, mean ± SD)	8.4 ± 11.4	7.9 ± 6.3	8.1 ± 9.1	0.421	0.028
Malapposed >400 µm (%, mean ± SD)	5.6 ± 10.3	8.1 ± 6.7	0.0 ± 0.0	0.189	0.028
Malapposed >400 µm and uncovered (N, mean ± SD)	6.7 ± 9.3	5.9 ± 4.2	6.3 ± 7.2	0.425	0.030
Malapposed >400 µm and uncovered (%, mean ± SD)	4.4 ± 8.0	5.9 ± 4.6	0.0 ± 0.0	0.176	0.005

dMB, distal main branch; LM, left main; POC, polygon of confluence; SD, standard deviation; NA, not applicable.

**Fig. 6.**
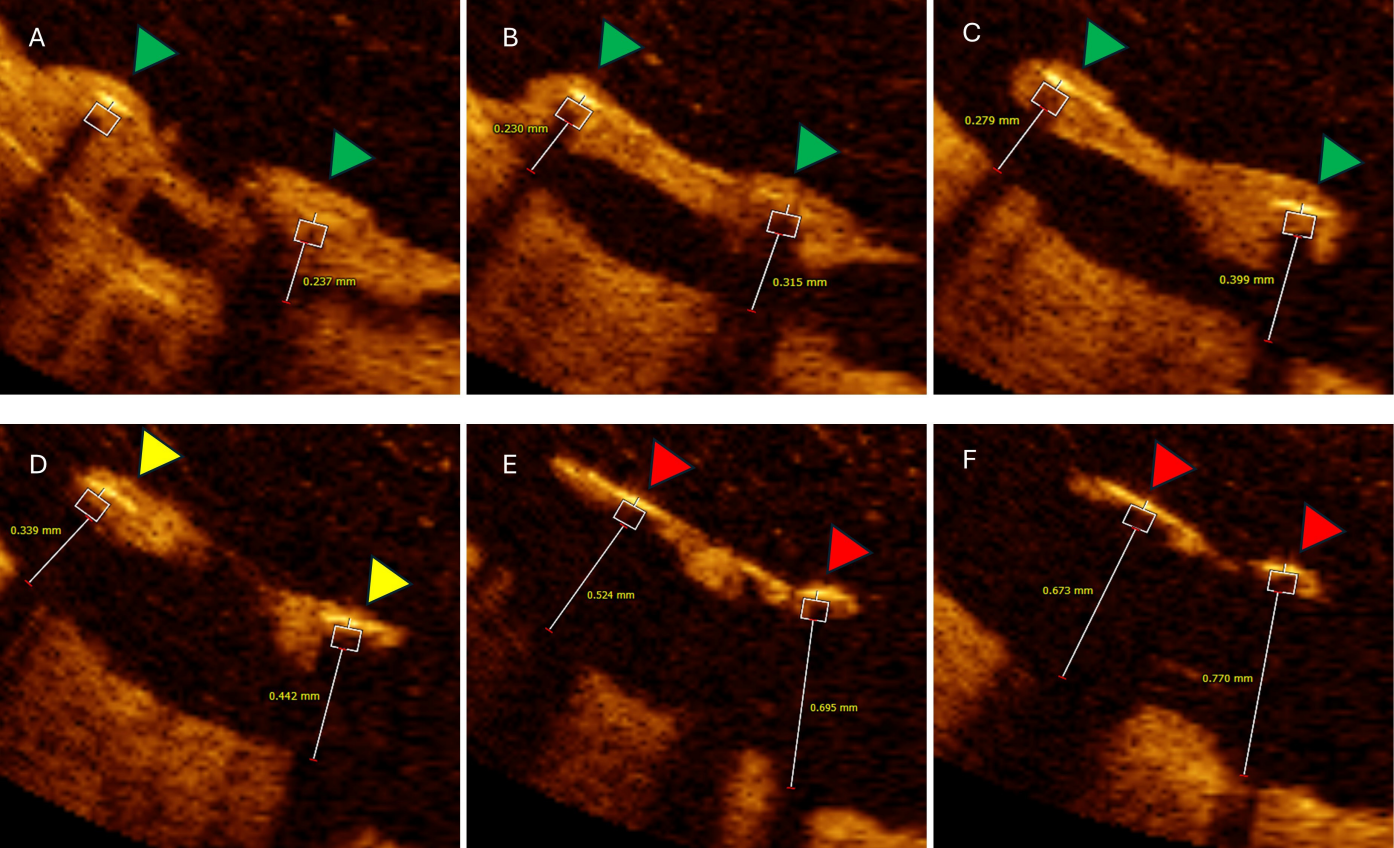
**Six consecutive OCT cross-sections illustrating impact of malapposed distance on follow-up neointimal coverage**. Analysed cross-section distance 0.2 mm. (A–C) Two malapposed but completely covered struts (green triangles) with hyperplastic tissue extensions from adjacent vessel wall with maximal malapposition distance bellow 400 µm. (D) Same malapposed but partially covered struts (yellow triangles). (E,F) Same malapposed and uncovered struts with measured malapposition distance >400 µm (red triangles). OCT, optical coherence tomography.

Malapposed struts with distance >400 µm were less likely to be covered by proliferating intimal hyperplasia compared to <400 µm malapposed struts (15.4 ± 21.6 vs. 24.8 ± 23.9%; *p* = 0.011) (Fig. 6).

### 3.5 Impact of Stent Optimization Techniques

Mean proximal reference diameter was 4.6 ± 0.9 mm and mean proximal reference area was 16.8 ± 4.6 mm^2^. Baseline POT was performed at the discretion of the operator in 12 (80.0%) cases with a mean balloon diameter of 4.4 ± 0.5 mm. POT only, was performed in 6 (40%), POT and KBI in 6 (40%) while in 3 patients (20%) neither was performed (Table 5). POT and POT+KBI were performed in patients with numerically larger proximal reference areas compared to patient without stent optimization (17.2 ± 2.7 vs. 17.1 ± 6.2 vs. 13.1 ± 4.1 mm^2^; *p* = 0.207). As expected, performing POT or POT+KBI resulted in larger mean stent area in the LM (13.9 ± 3.07 vs. 14.8 ± 3.4 vs. 8.5 ± 0.6 mm^2^; *p* = 0.030) and POC segment (11.9 ± 2.5 vs. 12.8 ± 5.6 vs. 9.6 ± 1.0 mm^2^; *p* = 0.564) compared to patients without stent optimisation. However, difference in strut coverage was not observed in the POT or POT+KBI groups in LM (86.5 ± 9.1 vs. 78.4 ± 21.5 vs. 78.6 ± 22.4%; *p* = 0.700) or the POC segment (83.1 ± 8.5 vs. 85.8 ± 10.5 vs. 80.5 ± 10.5%; *p* = 0.746), compared to patients with no additional optimization steps. The performance of POT+KBI resulted in a significantly reduced absolute number of malapposed struts and mean malapposition area in only the POC segment (Table 5).

**Table 5.** **Quantitative OCT analysis of stent optimization techniques**.

	LM segment				POC segment			
	POT only	POT+KBI	No optimization	*p*	POT only	POT+KBI	No optimization	*p*
	N = 6	N = 6	N = 3		N = 6	N = 6	N = 3	
Reference area (mm^2^, mean ± SD)	17.2 ± 2.7	17.1 ± 6.2	13.1 ± 4.1	0.207	17.2 ± 2.7	17.1 ± 6.2	13.1 ± 4.1	0.207
MLA (mm^2^, mean ± SD)	10.1 ± 4.9	12.3 ± 6.2	6.9 ± 1.7	0.376	10.1 ± 2.8	10.4 ± 5.6	7.9 ± 0.6	0.682
Mean lumen area (mm^2^, mean ± SD)	12.9 ± 3.0	14.9 ± 6.1	8.1 ± 1.1	0.136	13.1 ± 3.1	13.2 ± 5.1	10.8 ± 0.6	0.662
Mean minimal lumen diameter (mm, mean ± SD)	3.5 ± 0.4	3.8 ± 0.8	2.9 ± 0.28	0.131	3.0 ± 0.4	2.7 ± 0.6	2.7 ± 0.4	0.436
Lumen area stenosis (%, mean ± SD)	41.6 ± 24.6	26.7 ± 21.6	59.2 ± 4.1	0.131	39.5 ± 19.6	34.1 ± 33.7	35.7 ± 20.1	0.944
Lumen volume (mm^3^, mean ± SD)	75.8 ± 43.1	75.2 ± 39.0	74.3 ± 68.7	0.999	33.9 ± 13.9	27.4 ± 17.3	33.5 ± 11.7	0.747
Stent volume (mm^3^, mean ± SD)	83.3 ± 56.1	78.3 ± 43.1	75.6 ± 62.3	0.964	27.8 ± 9.8	26.4 ± 17.7	28.3 ± 8.7	0.977
In-stent NIH volume (mm^3^, mean ± SD)	14.3 ± 13.8	8.9 ± 7.4	7.2 ± 4.9	0.555	4.6 ± 2.2	2.9 ± 1.7	3.1 ± 1.5	0.311
NIH area at MLA (mm^2^, mean ± SD)	2.5 ± 1.5	1.4 ± 0.5	1.6 ± 0.8	0.236	2.2 ± 1.0	1.9 ± 0.8	1.1 ± 0.3	0.204
Mean NIH area (mm^2^, mean ± SD)	2.0 ± 0.7	1.5 ± 0.8	0.9 ± 0.3	0.123	2.1 ± 1.1	1.6 ± 0.9	1.0 ± 0.2	0.189
Malapposition volume (mm^3^, mean ± SD)	3.5 ± 3.5	3.0 ± 3.8	4.1 ± 5.9	0.937	8.3 ± 3.4	2.9 ± 1.7	7.1 ± 5.1	0.050
Mean malapposition area (mm^2^, mean ± SD)	2.4 ± 3.7	1.6 ± 2.8	0.3 ± 0.3	0.613	3.2 ± 1.3	0.8 ± 0.9	2.1 ± 1.1	0.013
Mean stent area (mm^2^, mean ± SD)	13.9 ± 3.07	14.8 ± 3.4	8.5 ± 0.6	0.030	11.9 ± 2.5	12.8 ± 5.6	9.6 ± 1.0	0.564
Stent area at MLA (mm^2^, mean ± SD)	11.7 ± 4.3	12.8 ± 4.3	8.3 ± 1.3	0.317	11.6 ± 1.6	12.0 ± 4.9	8.6 ± 0.9	0.394
Minimal stent area (mm^2^, mean ± SD)	10.9 ± 4.6	10.9 ± 2.9	7.5 ± 1.0	0.341	9.9 ± 0.5	7.9 ± 2.3	8.1 ± 0.3	0.682
Minimal stent expansion (%, mean ± SD)	64.0 ± 22.0	72.8 ± 31.9	45.3 ± 9.5	0.340	58.9 ± 11.8	50.1 ± 13.7	67.1 ± 21.9	0.293
Difference reference area - mean stent area (mm^2^, mean ± SD)	3.1 ± 2.9	2.3 ± 3.7	8.8 ± 4.9	0.067	5.3 ± 2.4	4.4 ± 2.7	3.5 ± 3.4	0.648
Covered (%, mean ± SD)	14.9 ± 12.9	75.9 ± 24.5	78.3 ± 23.3	0.863	73.9 ± 8.3	84.2 ± 11.4	80.5 ± 10.5	0.289
Uncovered (%, mean ± SD)	6.0 ± 3.8	10.7 ± 9.8	14.9 ± 12.7	0.349	6.7 ± 4.3	7.3 ± 6.6	7.1 ± 4.1	0.754
Malapposed (N, mean ± SD)	19.8 ± 25.8	14.3 ± 19.3	57.7 ± 99.0	0.405	21.7 ± 8.7	6.5 ± 5.6	12.0 ± 6.0	0.140
Malapposed (%, mean ± SD)	11.6 ± 15.1	11.3 ± 16.6	6.7 ± 10.7	0.871	19.3 ± 6.1	8.5 ± 7.9	12.4 ± 7.5	0.085
Malapposed and uncovered (%, mean ± SD)	7.4 ± 9.4	10.8 ± 18.6	6.4 ± 10.1	0.879	10.1 ± 5.7	6.9 ± 6.5	12.4 ± 7.5	0.479
Malapposition >400 µm (N, mean ± SD)	19.8 ± 25.8	14.3 ± 19.3	57.6 ± 99.0	0.972	9.0 ± 3.4	3.3 ± 3.5	6.0 ± 4.3	0.007
Malapposition >400 µm (%, mean ± SD)	4.7 ± 6.7	8.8 ± 15.1	0.9 ± 1.6	0.571	13.6 ± 6.6	4.9 ± 5.7	5.6 ± 2.9	0.066
Malapposition >400 µm and uncovered (%, mean ± SD)	4.1 ± 6.2	7.1 ± 11.9	0.9 ± 1.6	0.596	8.4 ± 3.8	4.5 ± 5.4	5.6 ± 2.9	0.392
Total covered (covered + malapposed covered) (%, mean ± SD)	86.5 ± 9.1	78.4 ± 21.5	78.6 ± 22.4	0.700	83.1 ± 8.5	85.8 ± 10.5	80.5 ± 10.5	0.746

LM, left main; POC, polygon of confluence; POT, proximal optimization technique; KBI, kissing balloon inflation; MLA, minimal lumen area; NIH, neointimal hyperplasia; OCT, optical coherence tomography; SD, standard deviation.

## 4. Discussion

The present analysis provides OCT insights into the late imaging results of DES implantation during the primary PCI of culprit ULM bifurcation lesions. The key findings are: (1) Regarding the primary endpoint, the strut coverage is not optimal in proximal bifurcation segments; (2) Although contemporary stent optimization procedures were performed, late malapposition and stent under-expansion were common because of the disproportionately larger proximal bifurcation segments compared to the DES used; (3) Strut malapposition distance affects strut endothelization during long-term follow-up; (4) Invasive imaging revealed a high incidence of subclinical de-novo atherosclerosis and restenotic intimal hyperplasia, necessitating late optimization and additional treatment.

PCI of bifurcation lesions is more likely to result in death, myocardial infarction, and repeat revascularization compared to non-bifurcation PCI [17]. During bifurcation PCI, complex stent implantation techniques are often required, in which the stent must be specifically adapted to the underlying anatomy [18, 19]. Early pathological post-mortem analyses indicated that bifurcation stenting is a significant risk factor for ST due to the presence of uncovered and malapposed struts [20]. However, these analyses didn’t identify the extent or pattern of strut coverage for stents deployed at the bifurcation. In our patient population, we demonstrated a persistent suboptimal level of strut coverage in left main bifurcation lesions, particularly in large proximal portions (78.9 and 79.8% for LM and POC, respectively).

Our findings are in line with previous reports that demonstrated, albeit at earlier time points, higher rates of incomplete endothelialization, also limited to the proximal bifurcation segments [10, 12, 21]. In the largest reported serial OCT analysis of 33 patients undergoing elective ULM PCI with DES, Fujino *et al*. [10] reported relatively high rates of uncovered and malapposed struts (18.7% and 5.3%, respectively) in the LM region at 9 months. Although, the percentage of malapposed struts decreased in follow-up compared to baseline (5.3% vs. 13.9%; *p* < 0.001), it still demonstrates that in larger bifurcation segments, acute, and persistent malapposition represents a frequent and clinically important finding, which impacts strut coverage and proper device endothelization. Our long-term analysis with observed rates of malapposed (11.7%) and uncovered struts (9.7%) in the proximal LM segment, confirms that these adverse findings persist beyond the 1-year period, which highlights the importance of prolonged duration of a dual antiplatelet regimen following ULM PCI in acute coronary syndrome (ACS). Since prior OCT analyses of patients with late ST showed a significant amount of uncovered (46%) and malapposed struts (39–42%), patients with suboptimal DES healing may be more susceptible to late ST and other adverse cardiovascular events [22, 23, 24].

If acute stent malapposition is not corrected, it decreases over time as a result of neointimal proliferation, and the distance of the strut to the vessel wall [25]. Serial OCT evaluations showed that endothelialization progresses rapidly in the early period after DES implantation reaching more than 90% at three months [26]. In our later analysis (average 1580 days), the time interval to follow-up OCT did not correlate with the percentage of struts covered (*p* = 0.343). Since this was not an imaging analysis with serial assessments of the same devices but only a very late observational evaluation, we could not investigate the relationship between strut coverage and the post-PCI time interval.

In our analysis, significantly malapposed struts >400 µm were less likely to be covered by proliferating intimal hyperplasia compared to <400 µm malapposed struts (15.4 ± 21.6 vs. 24.8 ± 23.9%; *p* = 0.011). Previous reports have shown that stent endothelialization is significantly influenced by the distance of the malapposed struts from the vessel wall: the greater the distance of acute malapposition, the greater the likelihood of persistent malapposition during follow-up and delayed healing [27]. In contrast, struts closer to the vessel wall, although still malapposed, may undergo endothelialization to a greater extent over time (Fig. 4) [28, 29]. Therefore, the risk of late ST is significantly increased in struts with severe malposition (≥400 µm) because they often have incomplete, delayed or absent endothelialization [30]. Therefore, to promote long-term healing and reduce the risk of late complications, our results, along with prior reports, emphasize the importance of achieving optimal acute stent apposition, or the smallest achievable malapposition distance, during the initial procedure. Notably, neither on OCT nor during follow-up, was there any evidence of recent subclinical thrombi formation or clinically apparent ST, even though larger portions of the ULM had a high rate of uncovered and malpositioned stent struts. Two patients showed evidence of organized thrombi on overhanging, uncovered struts (one case with large metallic neocarina after TAP stent implantation). However, our small sample size precludes drawing definitive conclusions about this clinically important but relatively rare adverse event.

All baseline primary PCI procedures were performed by experienced operators in a high-volume primary PCI center (around 800 primary PCIs and 100 ULM PCIs annually during the study period). POT was performed with angiographically, 1:1 sized, non-compliant or semi-compliant balloons at high pressures. However, high rates of late malapposition were still noted in the LM and POC regions due to the disproportionately larger proximal reference size, highlighting the importance of balloon sizing according to IVI measurements and achieving a larger stent area, in contrast to the results of angiographic balloon sizing reported in this study. We found that larger reference area to mean stent area mismatch, was associated with the percentage of total covered struts (R^2^ = 0.268, *p* < 0.001), significantly malapposed >400 µm (R^2^ = 0.189, *p* = 0.028), and significantly malapposed >400 µm and uncovered struts (R^2^ = 0.176, *p* = 0.005), across bifurcation segments. The importance of IVI device sizing, as suggested by consensus documents, was reported in the clinical setting in the prospective LEMON study [31]. OCT-guided PCIs of ULM lesions were found to be feasible and provided overall positive study results, with stent expansion reaching 94% for the proximal LM segment (compared to 63% in our population) and a low significant malapposition rate in 18% of cases. However, whether OCT guidance can improve the acute and long-term outcomes of ULM PCI compared with angio-guided treatment remains to be elucidated. Currently, the advantage of OCT-guided bifurcation PCI was recently demonstrated in the randomized, outcomes-oriented OCTOBER trial, which demonstrated a lower incidence of adverse events in the OCT-guided group at 2 years [32]. Patients undergoing PCI for a ULM lesion represented a significant proportion of the population studied and contributed to the overall positive outcomes, although subgroup analysis showed no beneficial effect for this type of lesion.

Finally, five patients (33.3%) underwent ad hoc OCT-guided ULM reintervention due to subclinical DES failure. According to the collected data, DES is effective in preventing early and mid-term restenosis, but are unable to absolutely prevent proliferative neointimal hyperplasia and the development of neo-atherosclerosis, especially if not properly expanded or adequately sized [33, 34]. Specifically, for the ULM PCI, Kang *et al*. [34] showed that stent underexpansion detected by IVI, results in lower event-free survival at 9 months. The minimal stent area (MSA) cutoffs that best predicted restenosis was 5.0 mm^2^ for ostial left circumflex, 6.3 mm^2^ for ostial left anterior descending (LAD), 7.2 mm^2^ for the POC, and 8.2 mm^2^ for the LM segment. Although measured MSA in our population was above the specified criteria (10.3 mm^2^ for LM and 7.1 mm^2^ for LAD), neoatheroslerosis was detected in 33.3% of cases at long-term follow-up. According to the published data, neoatherosclerosis is one of the leading underlying reasons for very-late ST, reported to be present in 22–31% of late events [24, 35]. It is well known that coronary bifurcation lesions have a higher risk of developing in-stent restenosis than non-bifurcation lesions [17]. This is partially due to distinct flow patterns and areas of low shear stress [36]. Finally, there is insufficient evidence regarding the optimal treatment strategy for stent failure in ULM, as surgical revascularization is usually considered due to the unique challenges associated with a failed PCI in ULM lesions [37]. Further research is necessary to confirm our findings.

## 5. Limitations

There are several limitations of this study. The main limitation is the observational design and small sample size, therefore, no definitive conclusions regarding the impact of the observed OCT findings on clinical outcomes is possible. This is a pilot study, and findings should be considered hypothesis-generating and need to be investigated in future larger studies. However, this study illustrates for the first time a very late vascular response after angio-guided implantation of DES during ULM primary PCI using high-resolution imaging and unintended stent deformation, which resulted in reinterventions in 33.3% of patients. Since IVI was not performed at baseline, it is not possible to compare very late follow-up imaging findings with the results of the index procedure. In addition, incomplete blood clearance at the LM ostium impaired adequate OCT assessment of the ostial segment. The current study population included only subjects who did not experience any clinical events and TLR during the follow-up, and excluded patients without severe renal and heart failure, which reduces the generalizability of our results.

## 6. Conclusions

Long-term OCT evaluation of coronary stents implanted during primary PCI for ULM lesions demonstrates high rates of incomplete strut coverage, late malapposition, de novo atherosclerosis, and restenotic neointimal hyperplasia. The unfavourable OCT results of angio-guided DES implantations advocate for attention and greater implementation of image optimization strategies during primary PCI. Further research is needed to validate these results and improve the percutaneous treatment of ULM lesions during primary PCI.

## Data Availability

The datasets used and/or analyzed during the current study are available from the corresponding author on reasonable request.

## Abbreviations

DES, drug-eluting stents; DKC, double kissing crush; CABG, coronary artery bypass graft; CKF, chronic kidney failure; EF, ejection fraction; FMC, first medical contact; HF, heart failure; IVI, intravascular imaging; KBI, kissing balloon inflation; LAD, left anterior descending; LM, left main; MADS, Main, Across, Distal, Side; MB, main branch; MSA, minimal stent area; NIH, neointimal hyperplasia; Non-TLR, non-target lesion revascularization; OCT, optical coherence tomography; PCI, percutaneous coronary intervention; POC, polygon of confluence; POT, proximal optimization technique; SB, side branch; SD, standard deviation; ST, stent thrombosis; STEMI, ST-elevation myocardial infarction; TAP, T-And Protrusion; TLR, target lesion revascularization; ULM, unprotected left main.

## Supplementary Material

Supplementary material associated with this article can be found, in the online version, at https://doi.org/10.31083/j.rcm2512445.
