# Supplementary material for: Long-term Follow-up Optical Coherence Tomography Assessment of Primary Percutaneous Coronary Intervention for Unprotected Left Main
Source: Rev Cardiovasc Med. 2024 Dec 19;25(12):445. doi: 10.31083/j.rcm2512445 (PMC11683725; doi:10.31083/j.rcm2512445)
Supplement: Supplementary file 1 [file 2153-8174-25-12-445-s1.docx]

| OCT variable | Definition |
| --- | --- |
| Stent length | Total number of frames between proximal and distal stent reference point* slice thickness (mm) |
| Proximal reference lumen area | The area bounded by the luminal border at the proximal reference point |
| Distal reference lumen area | The area bounded by the luminal border at the distal reference point |
| Minimal Lumen Area (MLA) | The area bounded by the luminal border at the point of maximal obstruction |
| Mean minimal lumen diameter | Average of minimal lumen diameters calculated for every frame within stented segment |
| Mean lumen area | Average of cross-sectional lumen areas calculated for every frame within stented segment |
| No. of analyzed struts per cross-section | Sum of all analyzed struts/number of analyzed cross-sections within stented segment |
| Covered stent strut | Struts with tissue (positive intimal thickness) completely covering strut luminal border |
| Uncovered stent strut | Struts in contact with lumen with absent/incomplete tissue coverage with of strut luminal border (intimal thickness <1 µm) |
| Malapposed stent strut | Struts not in contact with the lumen with measured distance greater than strut thickness + polymer thickness and less than <400 µm |
| Significantly malapposed stent strut | Struts not in contact with lumen with measured distance >400 µm |
| Minimal stent area | Smallest stent area within stented segment |
| Mean stent area | Sum of stent areas/No of frames with stent area measurements |
| In-stent lumen volume | Sum of all lumen areas within stent region*slice thickness (mm) |
| In-stent stent volume | Sum of all lumen areas within stented segment*slice thickness (mm) |
| Neo Intimal Hyperplasia (NIH) Volume | Amount of plaque within stent lumen area*slice thickness (mm) |
| Neo Intimal Obstruction percentage | ([NIH volume]/stent volume)*100 |
| Mean NIH area | Sum of NIH areas/No of NIH areas |
| Minimal stent expansion | Stent expansion is defined by the MSA measured in the proximal and distal stented segments/respective reference lumen areas*100. The stent length is divided into 2 segments (proximal and distal), the division was set at the level of carina. |
| Mean stent expansion | (Stent cross-sectional area divided by respective reference lumen area / number of cross-sectional stent areas)*100 |
| Thrombus | Intracoronary thrombus is identified as an intraluminal mass with *irregular contour* protruding into lumen ≥ 200 µm at the thickest point, connected or not to the intimal surface. In case of thrombus presence, further classified as white, red or organized according to their optical and characteristics. |
